# Substance P–expressing neurons in the hypothalamic paraventricular nucleus mediate chronic cough hypersensitivity via the hypothalamus–airway neural pathway

**DOI:** 10.3389/fnins.2026.1816238

**Published:** 2026-05-12

**Authors:** Xiaoyan Zhang, Jingyang Chen, Chun Lin, Tao Xiao, Bin Wu, Fei He

**Affiliations:** 1 Department of Epidemiology and Health Statistics, School of Public Health, Fujian Medical University, Fuzhou, China; 2 Department of Pediatrics, The First Affiliated Hospital of Fujian Medical University, Fuzhou, China; 3 Allergy Center, The First Affiliated Hospital of Fujian Medical University, Fuzhou, China; 4 Fujian Provincial Key Laboratory of Brain Aging and Neurodegenerative Diseases, School of Basic Medical Sciences, Pain Research Institute, Fujian Medical University, Fuzhou, Fujian, China; 5 Union Clinical Medicine College, Fujian Medical University, Fuzhou, Fujian, China

**Keywords:** cough hypersensitivity, guinea pig model, hypothalamic paraventricular nucleus, neural pathway, substance P

## Abstract

**Background and purpose:**

Increased cough sensitivity is the key pathophysiological mechanism of chronic cough. Although previous studies have focused on peripheral airway receptor sensitization, the role of the central nervous system—particularly the hypothalamic paraventricular nucleus (PVN)—remains unclear. Whether substance P (SP)–expressing PVN neurons contribute to cough hypersensitivity remains unknown.

**Methods:**

Three-week-old Hartley guinea pigs were divided into three groups: citric acid (CA), saline control (SA), and blank control (CON). A cough hypersensitivity model was induced by inhalation of 0.4 mol/L citric acid. Cough sensitivity was assessed using a capsaicin challenge, with the C5 threshold defined as the lowest capsaicin concentration inducing ≥5 coughs. Locomotor activity was evaluated using the open-field test. Airway inflammation and goblet cell hyperplasia were examined by HE and PAS staining. SP and c-Fos expression in the PVN were detected by immunofluorescence and Western blot. HSV retrograde tracing was used to analyze the PVN-airway neural pathway associated with cough hypersensitivity.

**Results:**

Compared with the control groups, guinea pigs in the CA group exhibited a time-dependent increase in cough frequency and enhanced cough sensitivity, as indicated by a reduction in the C5 threshold. Histological analysis revealed increased inflammatory cell infiltration and goblet cell hyperplasia in the airways of the CA group. SP and c-Fos expression, along with the proportion of SP/Fos double-labeled neurons in the PVN, were significantly increased in the CA group (all *P* < 0.05). Viral tracing confirmed the presence of HSV-positive neurons in the PVN, supporting a neural connection between the PVN and the airways.

**Conclusion:**

Activation of SP-expressing neurons in the PVN is associated with cough hypersensitivity and suggests the presence of a potential PVN–airway neural pathway. These findings provide a theoretical basis for the development of central-targeted therapies for chronic cough.

## 1 Introduction

Chronic cough is one of the common symptoms of pediatric respiratory diseases, and its key pathophysiological feature is an abnormally increased cough sensitivity (Chung et al., 2022). Clinically, this group of conditions is collectively described as cough hypersensitivity syndrome (CHS), which is characterized by a reduced cough reflex threshold whereby even mild stimuli can trigger intense coughing (Chung and Mazzone, 2026; Peters et al., 2025). Previous studies have shown that the levels of neuropeptides in the airways are significantly increased in patients with heightened cough sensitivity, among which substance P (SP) is particularly elevated (Cheng et al., 2021; Shi et al., 2019). These findings suggest that neuropeptide signaling pathways play an important role in the development of cough hypersensitivity. SP is a key neurotransmitter released from the terminals of unmyelinated C fibers and is widely involved in the transmission of peripheral sensory signals (Mashaghi et al., 2016).

Previous studies have mainly focused on the sensitization of peripheral airway receptors and sensory nerve endings (Drake et al., 2021; Shapiro et al., 2021). However, cough is a complete reflex arc, and the central nervous system, as an essential component of this reflex pathway, may also participate in the regulation of cough sensitivity (Lu and Cao, 2023). The nucleus tractus solitarius (NTS) in the medulla oblongata serves as the primary integrative center for receiving and processing airway sensory information. Within this nucleus, second-order neurons form synaptic connections with primary sensory afferent fibers transmitting cough-related signals. When sensory nerve terminals release neurotransmitters such as SP or glutamate, the excitability of second-order neurons can be enhanced, thereby promoting the central sensitization of the cough reflex (Cinelli et al., 2023; Gannot et al., 2024; Lu et al., 2024; Lucanska et al., 2020; Poliacek et al., 2017). The hypothalamic paraventricular nucleus (PVN), a higher-order center of the NTS, plays a critical role in neuroendocrine regulation and autonomic integration, and it sends descending projections to brainstem regions including the NTS (Iremonger and Power, 2025; Jiang et al., 2019; Ruyle et al., 2023; Savić et al., 2022). Previous studies have suggested that chronic inflammatory stimulation may lead to an upregulation of SP expression in the PVN (Chowdrey et al., 1995). In addition, the PVN has been shown to interact with respiratory control networks during various processes such as stress responses, pain modulation, and autonomic regulation (Bi et al., 2026; Ruyle et al., 2023). Although accumulating evidence indicates that the PVN participates in respiratory central regulation, its specific role in the development of cough hypersensitivity remains largely unclear, particularly in juvenile animal models (Liu et al., 2025; Tenorio-Lopes and Kinkead, 2021). This knowledge gap is especially prominent in pediatric chronic cough, as both the respiratory system and the central nervous system are still undergoing developmental maturation in children, highlighting the need for a deeper understanding of central sensitization mechanisms.

Based on the above background, we proposed the hypothesis that repeated airway stimulation may activate SP–expressing neurons in the PVN and promote the development of cough hypersensitivity through a PVN–airway neural pathway. To test this hypothesis, a cough hypersensitivity model was established in juvenile guinea pigs. A combination of immunofluorescence staining, Western blotting, and viral tracing techniques was employed to examine the expression of SP and Fos proteins in the PVN, the distribution of SP/Fos double-labeled neurons, and the neural connections between the PVN and the airway. The present study aimed to elucidate the role of the PVN–airway pathway in the regulation of cough sensitivity and to provide experimental evidence for potential central-targeted therapeutic strategies for chronic cough in children.

## 2 Materials and methods

### 2.1 Animals and experimental design

Twenty-eight 3-week-old male specific-pathogen-free (SPF) Hartley guinea pigs (200–250 g) were obtained from Zhejiang Jiashan Shengwang Ecological Farm [License No. SCXK (Zhejiang) 2023-0010]. All procedures were approved by the Institutional Animal Care and Use Committee and complied with international animal welfare guidelines.

Animals were housed in an SPF-grade barrier facility (25 °C ± 2 °C, 55% ± 10% humidity, 12 h light/dark cycle) with autoclaved bedding, free access to food and water, and two per cage, and were acclimated for 3 days before experiments.

Prior to cough testing, animals were pre-screened by exposure to aerosolized 0.4 mol/L citric acid for 10 min to assess induced cough responsiveness. Only animals exhibiting a cough frequency of 3–20 coughs/min were included. Four animals were excluded from further analysis, including two low responders (<3 coughs/min) and two high responders (>20 coughs/min). Consequently, 24 animals were enrolled in the subsequent experiments (Mashaghi et al., 2016).

**FIGURE 1:**
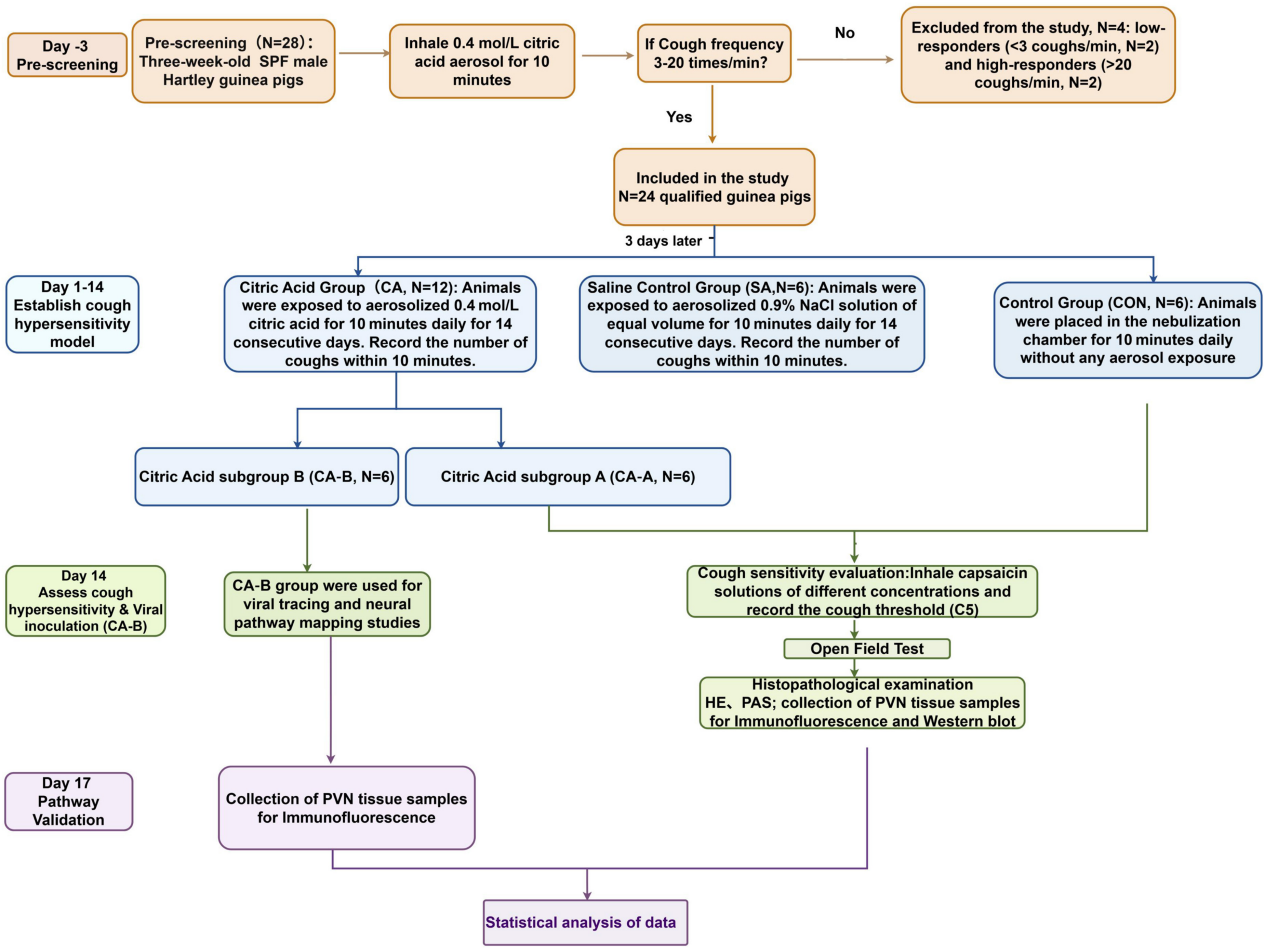
Schematic diagram of the experimental protocol.

The 24 prescreened guinea pigs were randomly divided into three groups: Citric Acid group (CA, *n* = 12): Animals were exposed to 0.4 mol/L citric acid aerosol for 10 min daily over 14 days to induce cough hypersensitivity (Drake et al., 2021). Saline control group (SA, *n* = 6): Animals received a 0.9% NaCl aerosol under the same conditions to control for mechanical and solvent effects. Blank control group (CON, *n* = 6): Animals were placed in the chamber without aerosol exposure as a baseline control. Post-experiment, the CA group was split into two subgroups (*n* = 6 each): CA-A for behavioral tests and molecular analyses, and CA-B for viral tracing and neural mapping (Figure 1).

### 2.2 Cough sensitivity assessment

Cough sensitivity was evaluated using a capsaicin cough challenge test. Capsaicin was diluted in saline to create concentrations from 10^–^8 to 10^–3^ mol/L, labeled 1 to 6. Conscious guinea pigs in an observation chamber were exposed to each concentration for 2 min, starting from the lowest. Cough frequency was recorded for 3 min after each exposure, with a 5-min recovery interval between tests (Shapiro et al., 2021). The cough threshold (C5) was the lowest capsaicin concentration causing at least five coughs, with a lower C5 indicating higher sensitivity (Lu and Cao, 2023). A multimodal monitoring system was used to distinguish coughs from similar behaviors.

**FIGURE 2:**
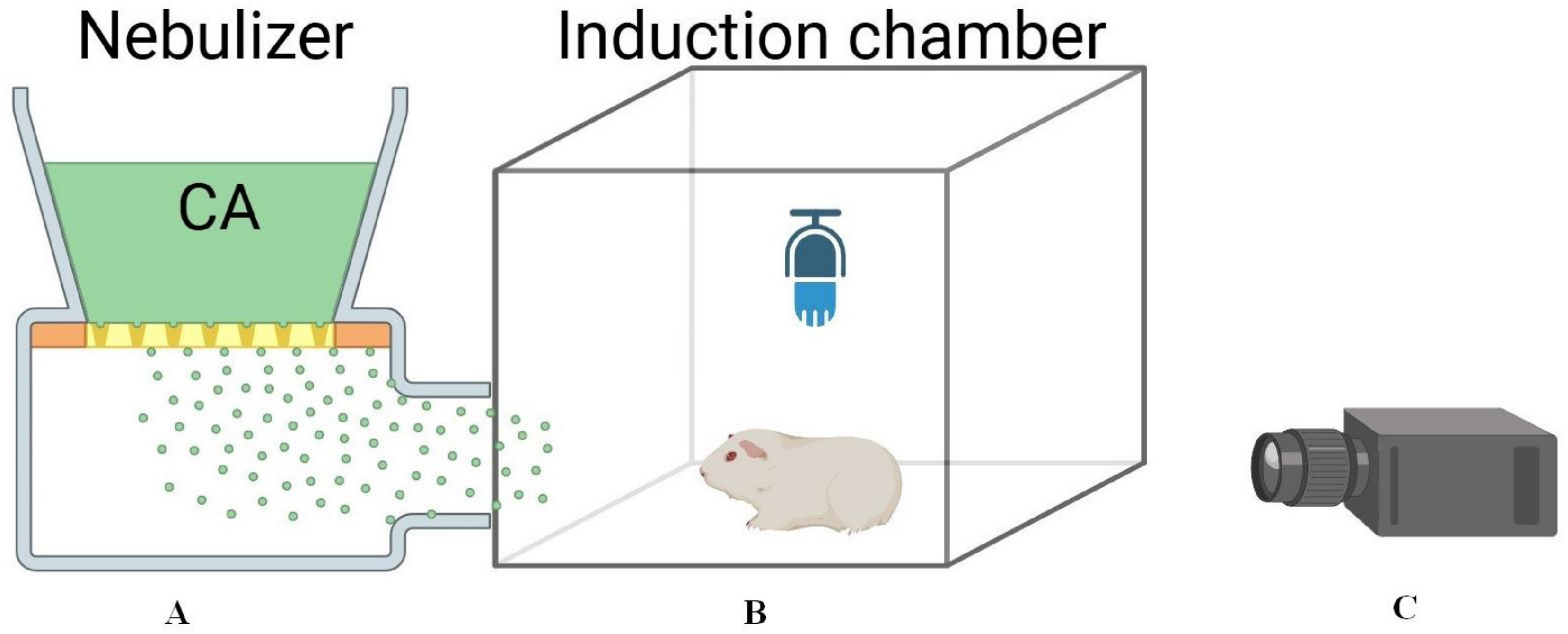
Multimodal cough monitoring system layout. **(A)** Ultrasonic nebulizer. **(B)** Induction chamber with directional mic inside. **(C)** HD camera at observation window for synchronized video-audio recording.

To accurately discriminate coughs from cough-like behaviors (e.g., sneezing, rapid breathing), we used a multimodal monitoring system for real-time recording (Figure 2). This system comprised: (A) an ultrasonic nebulizer (Model NE-U17, Omron Corporation, Japan) producing aerosol particles with a median diameter of 3.5 μm; (B) a high-definition video camera (Sony HDR-CX405, Sony, Japan) set at 60 fps and 1,920 × 1,080 resolution, positioned in front of a transparent observation chamber (40 cm × 40 cm × 40 cm); and (C) a directional condenser microphone (DJI Mic 2, DJI Technology, China) with a sampling rate of 48 kHz and sensitivity of −2, DJI Technology, China) with a sampling rate of 48 kHz and sensitivity of a sampling rate of 48 analysis.

Coughs and sneezes were distinguished based on the combination of video and auditory criteria (Li et al., 2021): coughs were identified by sudden abdominal contraction, mouth opening, neck extension, and forelimb lifting lasting 0.2–0.5 s, accompanied by short explosive sounds distinct from background noise; sneezes by rapid head movement with nasal discharge, forward body motion, and sharp high-pitched bursts often followed by an inspiratory phase. Each animal’s cough count was independently determined by two investigators blinded to group assignment. Final cough frequency was calculated as the mean of the two counts confirming high reliability.

### 2.3 Open-field test

The primary purpose of the open-field test in this study was to serve as a critical methodological control to exclude potential effects of the experimental intervention (repeated citric acid nebulization) on the spontaneous locomotor activity of the experimental animals (juvenile guinea pigs). This test is a well-established method in behavioral neuroscience for assessing the effects of experimental manipulations on spontaneous activity and anxiety-like behaviors (Kraeuter et al., 2019).

The specific procedure was as follows: after the completion of the entire cough model induction protocol, each guinea pig was individually placed in the center of the floor of an open-field arena (40 cm × 40 cm × 40 cm) and allowed to explore freely for 10 min. The total distance traveled by the guinea pig was measured using open field test software to reflect the overall activity level of the juvenile animals, thereby eliminating potential confounding factors arising from altered locomotor ability that might influence the assessment of cough sensitivity (Noble et al., 2017).

### 2.4 Immunohistochemistry and immunofluorescence

To accurately capture the peak expression of c-Fos protein, which typically occurs 60–90 min after neuronal activation, all animals underwent perfusion fixation within this critical time window following behavioral testing. Guinea pigs were anesthetized with sodium pentobarbital (40 mg/kg, i.p.) and transcardially perfused with saline followed by 4% paraformaldehyde in PBS. Brains were removed, hypothalamic region dissected, and tracheal tissues collected. For cryosectioning, brain tissues were dehydrated in 30% sucrose, embedded in OCT compound, and coronally sectioned at 20 μm.

For histopathological analysis (Zhao et al., 2026), selected brain and tracheal tissues were fixed in 4% paraformaldehyde, dehydrated, cleared, and embedded in paraffin. Additional fresh tissues were snap-frozen in liquid nitrogen and stored at −80 °C for protein extraction.

Paraffin-embedded tracheal sections were deparaffinized and stained with hematoxylin–eosin (HE) for inflammatory infiltration and structural alterations, and with periodic acid–Schiff (PAS) to assess goblet cell hyperplasia and mucus secretion.

Immunofluorescence staining was performed on brain sections after antigen retrieval (sodium citrate–EDTA buffer) and blocking. Sections were incubated overnight at 4 °C with rabbit anti-c-Fos (Santa Cruz Biotechnology, USA, 1:1200) and rabbit anti-substance P (ImmunoStar, USA, 1:1200), followed by fluorophore-conjugated secondary antibodies (Jackson ImmunoResearch, 1:200) for 1 h at room temperature. Nuclei were counterstained with DAPI and mounted with antifade medium. Images were acquired using a confocal microscope, and mean optical density of c-Fos– and SP-positive signals in the PVN was quantified with ImageJ.

### 2.5 Western blot analysis

Total protein was extracted from PVN tissues and quantified by bicinchoninic acid (BCA) assay. Equal amounts of protein were separated via SDS–PAGE and transferred to polyvinylidene difluoride (PVDF) membranes. Membranes were blocked with 5% non-fat milk at room temperature, then incubated overnight at 4°C with rabbit anti-c-Fos (Santa Cruz Biotechnology, 1:800) and rabbit anti-GAPDH (Cell Signaling Technology, 1:3000). After washing, membranes were incubated with HRP-conjugated secondary antibodies (Jackson ImmunoResearch, 1:5000) for 1 h at room temperature. Protein bands were visualized by ECL, and band intensities were measured with Image Lab software and normalized to GAPDH.

### 2.6 HSV retrograde neural circuit tracing

**FIGURE 3:**
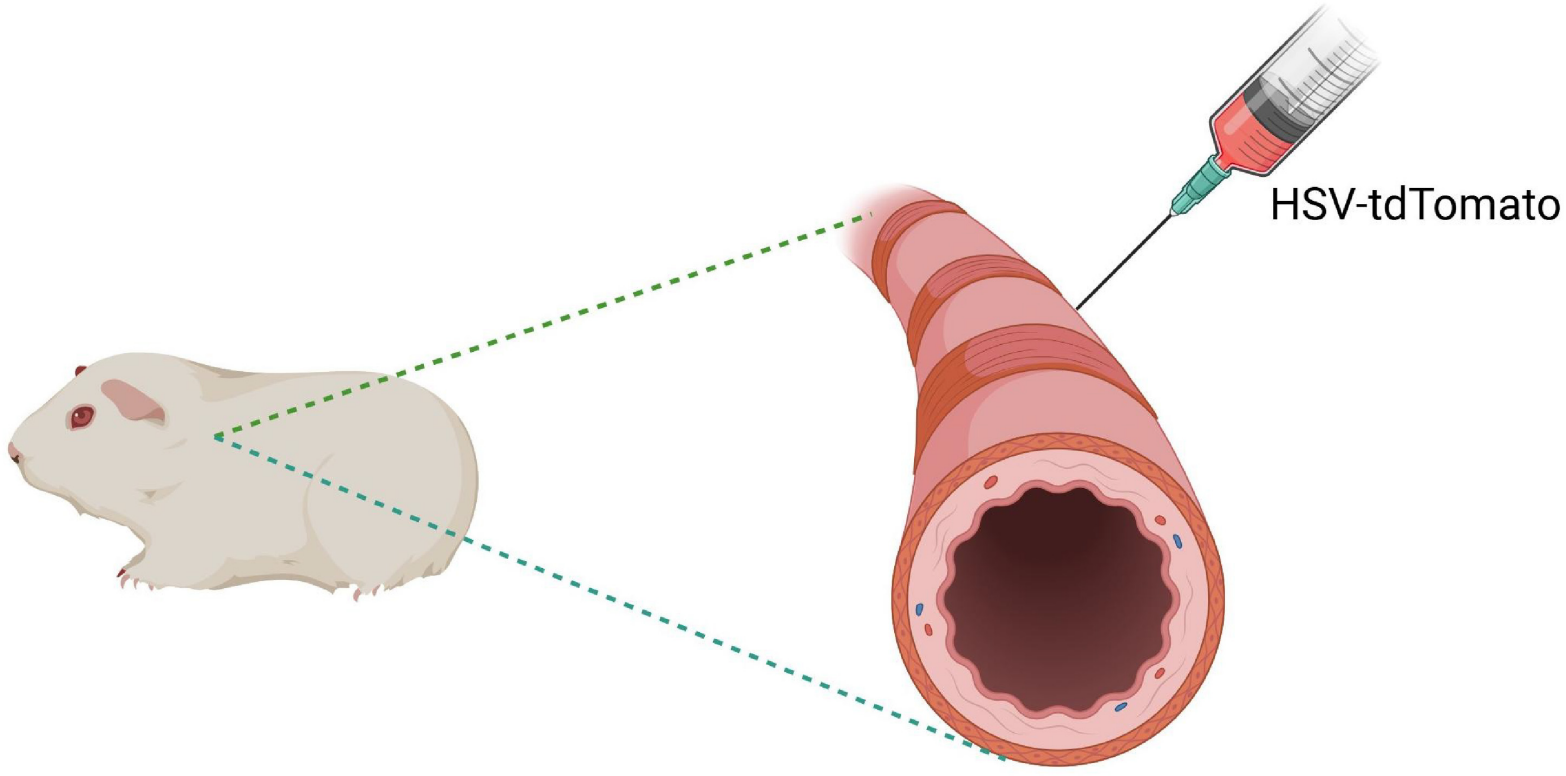
Schematic illustration of retrograde transsynaptic tracing using HSV-tdTomato.

To verify the neural connection between the PVN and the airway, retrograde transsynaptic tracing was performed using herpes simplex virus (HSV)-tdTomato (Wuhan Brain Science Technology Co., Ltd., Wuhan, China). Guinea pigs were anesthetized and placed in a supine position, and the cervical trachea was surgically exposed (Figure 3). A 34-gauge glass micropipette was used to inject 5 μL of HSV-tdTomato suspension into the tracheal adventitia at 3–5 sites. The needle was retained for 30 s after each injection before withdrawal, and the incision was then sutured (Cinelli et al., 2023; Lu et al., 2024).

Animals were allowed to survive for 72 h to permit viral transport. They were then transcardially perfused, and the brains were collected and sectioned. Virus-labeled neurons in the PVN were identified by immunofluorescence detection of tdTomato signals using an anti-tdTomato antibody (Origene, Rockville, MD, United States) to determine the presence of PVN–airway neural projections.

### 2.7 Statistical analysis

All data are expressed as mean ± SEM, unless otherwise specified. Statistical analyses were performed using GraphPad Prism 10.0 (GraphPad Software, San Diego, CA, United States). For normally distributed continuous variables with homogeneous variance, intergroup comparisons with >2 groups were analyzed by one-way ANOVA followed by Tukey’s *post-hoc* test for all pairwise comparisons. Repeated-measures ANOVA followed by Bonferroni *post hoc* correction was used to assess changes in cough frequency over time. Pairwise comparisons between two groups were performed using unpaired Student’s *t*-tests. Exact *P*-values, F- or t-statistics, degrees of freedom (df), and sample sizes (*n*) are reported for each comparison. A two-tailed *P* < 0.05 was considered statistically significant.

## 3 Results

### 3.1 Establishment of the citric acid–induced cough hypersensitivity model

To establish a stable cough hypersensitivity model, juvenile guinea pigs were exposed to daily citric acid aerosol inhalation for 14 consecutive days, followed by a systematic evaluation from multiple dimensions, including behavioral performance, cough reflex threshold, and locomotor activity.

#### 3.1.1 Changes in cough behavior

**FIGURE 4:**
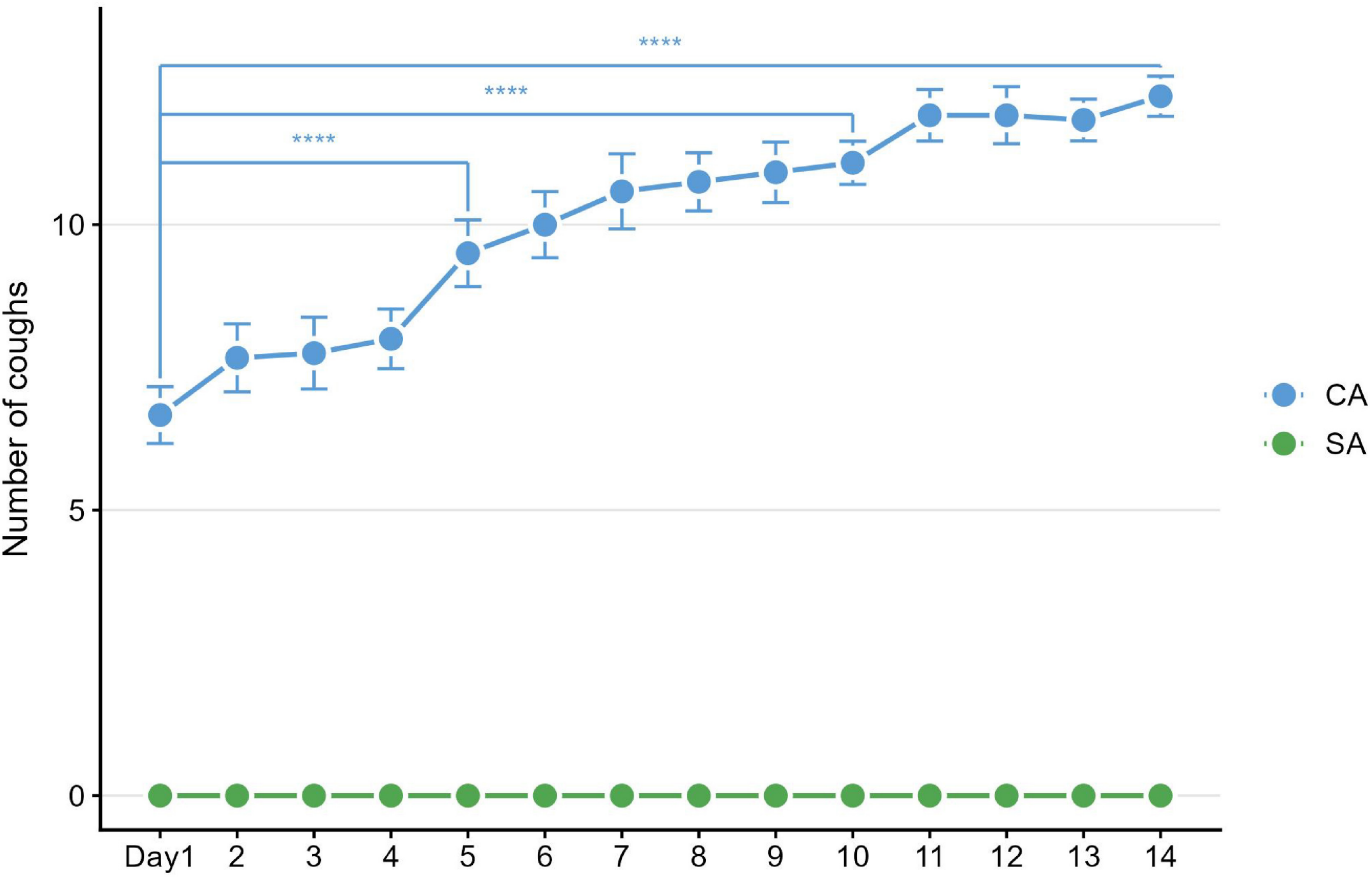
Dynamic changes in cough frequency (number of coughs per 10-min nebulization) in the citric acid (CA) and saline control (SA) groups over 14 days. Cough frequency was significantly higher in the CA group than in the SA group from day 5 to 14 (****P < 0.0001), with no detectable coughs in the SA group throughout the experiment.

Cough responses were monitored throughout the aerosol exposure period. Coughs were counted during the 10-min nebulization session. Compared with the SA group, the CA group showed a significant increase in cough frequency from day 5 to day 14, averaging 9.97 ± 0.30 coughs per 10 min over the 14-day period, based on mixed repeated-measures ANOVA, between-group effect: *F*(1, 16) = 323.35, *P* < 0.0001; CA *n* = 12, SA *n* = 6; Bonferroni *post hoc*: CA vs. SA at day 5, t(16) = 11.33, *P* < 0.0001; at day 10, t(16) = 20.39, *P* < 0.0001; at day 14, t(16) = 24.31, *P* < 0.0001. In contrast, the SA group showed no significant cough responses during the entire experimental period. These results demonstrate that repeated airway stimulation successfully induced a stable and sustained state of cough hypersensitivity (Figure 4).

#### 3.1.2 Reduced cough sensitivity threshold

**FIGURE 5:**
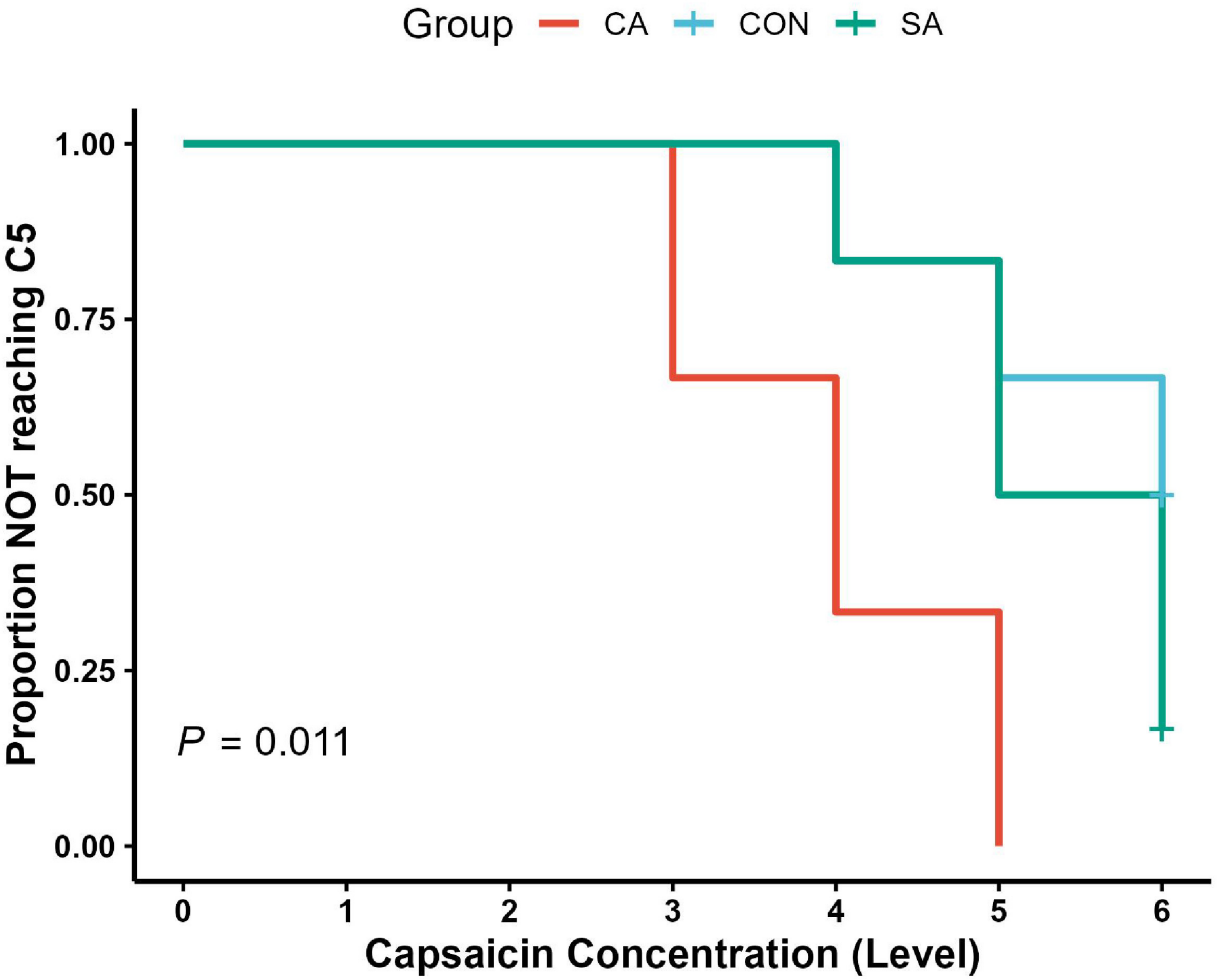
Kaplan-Meier survival curves illustrating the proportion of animals reaching the C5 cough threshold across increasing capsaicin concentrations. The citric acid (CA) group exhibited significantly lower C5 thresholds compared with the saline control (SA) and blank control (CON) groups (Kaplan–Meier, *P* = 0.011; *n* = 6 per group), indicating increased cough sensitivity.

To quantitatively evaluate changes in cough sensitivity, a capsaicin challenge test was conducted. In the CA group, the number of guinea pigs reaching the C5 threshold began to increase progressively starting from capsaicin concentration 3 (10^–^6 mol/L). In contrast, in the SA and CON groups, only a few individuals exhibited C5 responses even at the higher concentration 4 (10^–^5 mol/L). Kaplan-Meier survival analysis with Log-rank testing revealed a significant difference in cough threshold distribution among the three groups (χ^2^ = 9.1, df = 2, *P* = 0.011; *n* = 6 per group). *Post hoc* pairwise Log-rank comparisons (Benjamini-Hochberg correction) showed that the CA group reached the C5 threshold at significantly lower capsaicin concentrations than both the CON group (*P* = 0.039) and the SA group (*P* = 0.039), while no significant difference was observed between the CON and SA groups (*P* = 0.326), directly demonstrating a marked increase in cough reflex sensitivity in the model group (Figure 5).

#### 3.1.3 Assessment of locomotor activity by open-field test

**FIGURE 6:**
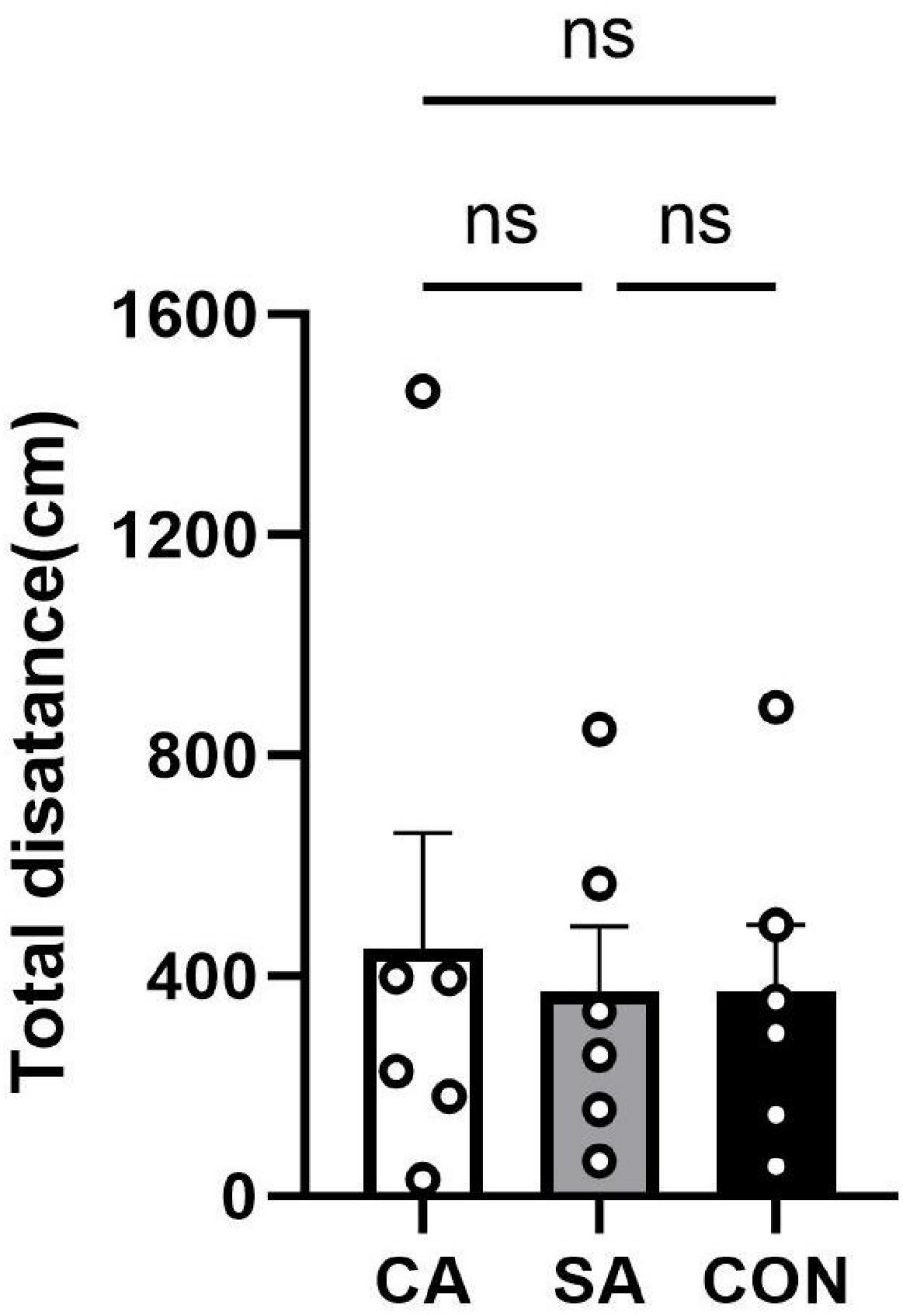
Results of the open-field test for assessing locomotor activity in each group.

To exclude potential confounding effects of altered locomotor activity on cough behavior, an open-field test was conducted to evaluate spontaneous activity levels. The results showed that there were no significant differences in total distance traveled among groups during the 10-min test [one-way ANOVA: *F* (2, 15) = 0.0862, *P* = 0.9178; CA *n* = 6, SA *n* = 6, CON *n* = 6; Tukey’s *post hoc*: CA vs. SA, *P* = 0.9400; CA vs. CON, *P* = 0.9989; SA vs. CON, *P* = 0.9240] (Figure 6), indicating that aerosol exposure did not significantly affect locomotor function and thereby ensuring the reliability of the cough sensitivity measurements.

### 3.2 Airway inflammation induced by citric acid

To explore the peripheral pathological mechanisms underlying cough hypersensitivity, histological analysis was performed on tracheal tissues from guinea pigs.

#### 3.2.1 Inflammatory cell infiltration

**FIGURE 7:**
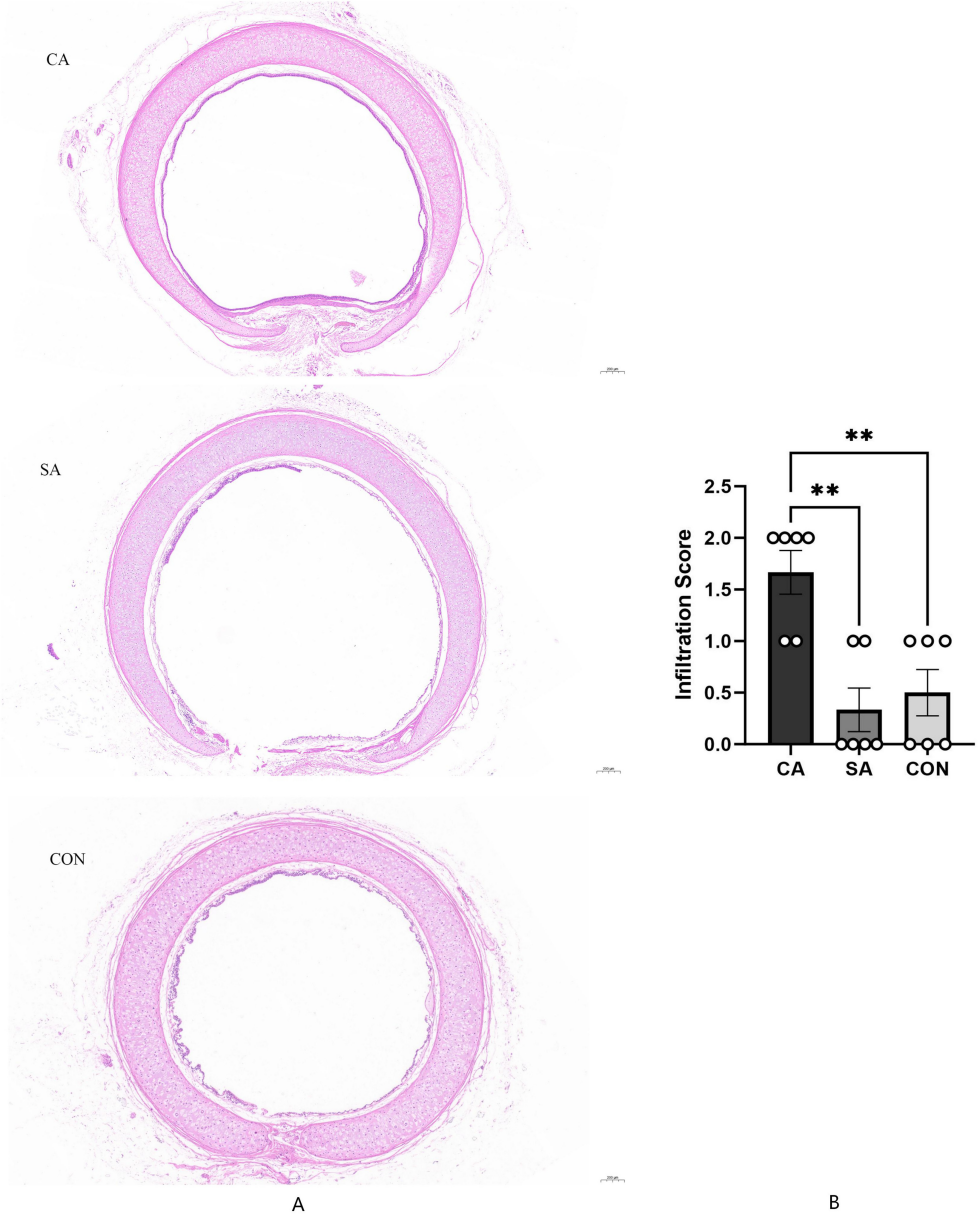
Hematoxylin–eosin (HE) staining of tracheal sections from the citric acid (CA), saline control (SA), and blank control (CON) groups. **(A)** Representative HE-stained transverse sections of trachea from the CA, SA, and CON groups, captured using an Olympus BX53 microscope (5 × objective). Scale bar = 200 μm. In the CA group, the tracheal epithelium showed mild disorganization, with prominent infiltration of inflammatory cells—primarily eosinophils—within the mucosal stroma, along with submucosal edema and dilation and congestion of small vessels. In contrast, the SA and CON groups exhibited well-preserved structural architecture, with orderly arranged epithelial layers and absence of significant inflammatory cell infiltration or tissue edema. **(B)** Semiquantitative scoring of inflammatory cell infiltration in tracheal tissues. Scoring criteria: 0, no infiltration; 1, mild infiltration in the airway lumen, mucosa, and submucosa; 2, multifocal inflammatory cell infiltration. Higher scores indicate more severe inflammation. Data are presented as mean ± SEM (*n* = 6 per group). Statistical analysis was performed using one-way ANOVA followed by Tukey’s *post-hoc* test; **P < 0.01 vs. CA group for both SA and CON groups.

Hematoxylin–eosin staining revealed that the tracheal epithelium in the CA group exhibited mild disorganization, with prominent infiltration of inflammatory cells, primarily eosinophils, within the mucosal stroma, along with submucosal edema and dilation and congestion of small vessels. In contrast, the tracheal tissues of the SA and CON groups retained intact structural architecture, with orderly arranged epithelial layers and no obvious inflammatory cell infiltration or tissue edema (Figure 7).

#### 3.2.2 Goblet cell hyperplasia and mucus secretion

**FIGURE 8:**
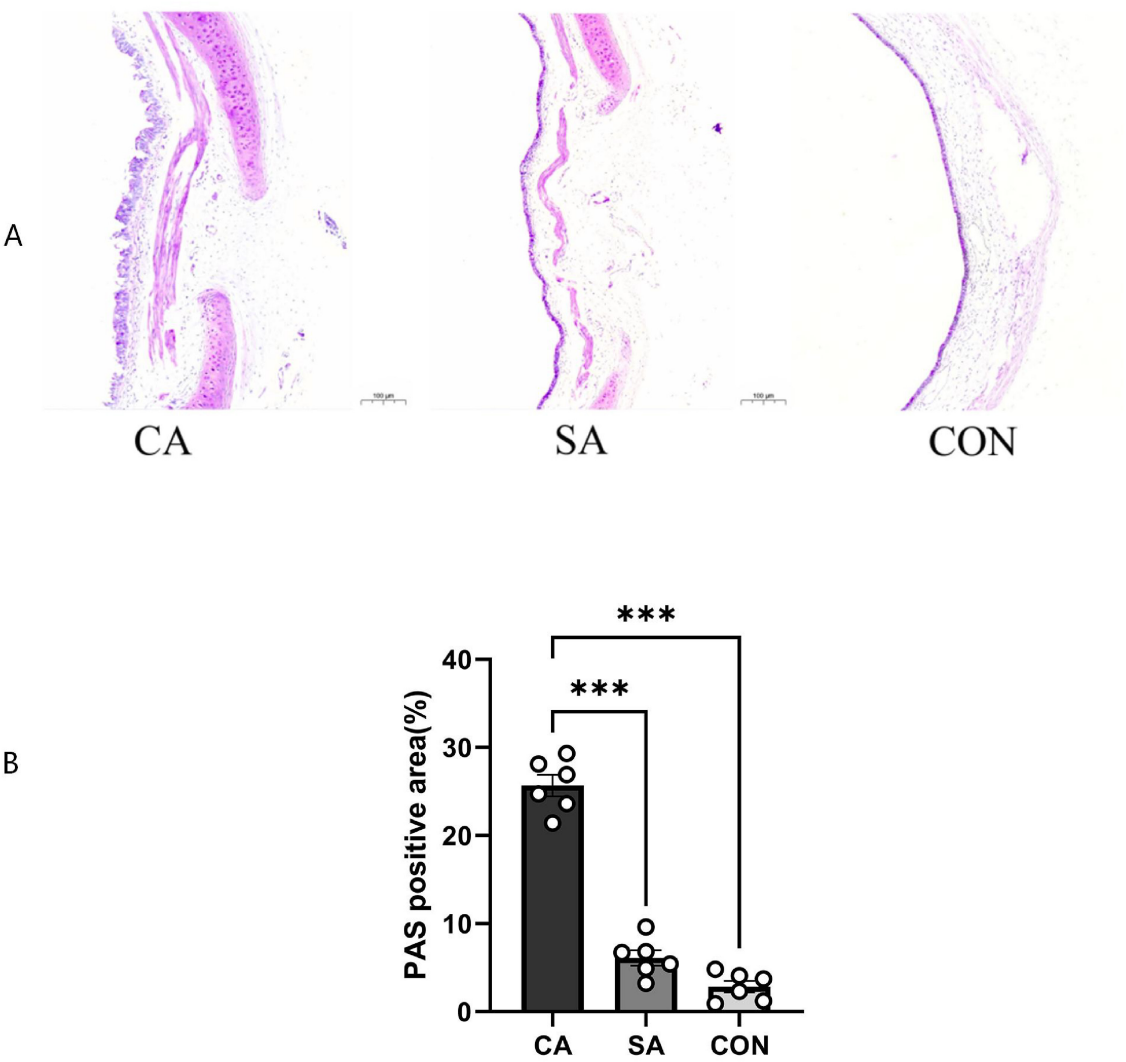
Periodic acid–Schiff (PAS) staining of tracheal sections from citric acid (CA), saline control (SA), and blank control (CON) groups. **(A)** Representative PAS-stained tracheal sections from the CA, SA, and CON groups, captured using an Olympus BX53 microscope equipped with a 10 × objective lens. Scale bar = 100 μm. In the CA group, marked airway remodeling was observed, characterized by extensive goblet cell hyperplasia and mucus plug formation within the airway lumen. In contrast, the SA and CON groups exhibited only sparse goblet cells, with no evidence of mucus retention or epithelial hyperplasia. **(B)** Semiquantitative analysis of PAS-positive area (%) in tracheal sections. Data are presented as mean ± SEM (*n* = 6 per group). Statistical analysis was performed using one-way ANOVA followed by Tukey’s *post-hoc* test; ***P < 0.001 vs. CA group (for both SA and CON groups).

Periodic acid–Schiff staining was used to assess goblet cell hyperplasia and mucus production in the airway epithelium. In the CA group, marked airway remodeling was observed, characterized by extensive goblet cell hyperplasia and mucus plug formation. In contrast, the SA and CON groups showed only sparse goblet cells, with no mucus retention in the airway lumen (Figure 8). Together with HE findings, these results indicate that repeated citric acid exposure induced chronic airway inflammation, featuring eosinophilic infiltration and goblet cell hyperplasia.

### 3.3 PVN neuron activation and neuropeptide upregulation in cough hypersensitivity

To clarify the central regulatory mechanisms of cough hypersensitivity, we analyzed neuronal activation and neuropeptide expression changes in the PVN.

#### 3.3.1 Increased c-Fos protein expression

**FIGURE 9:**
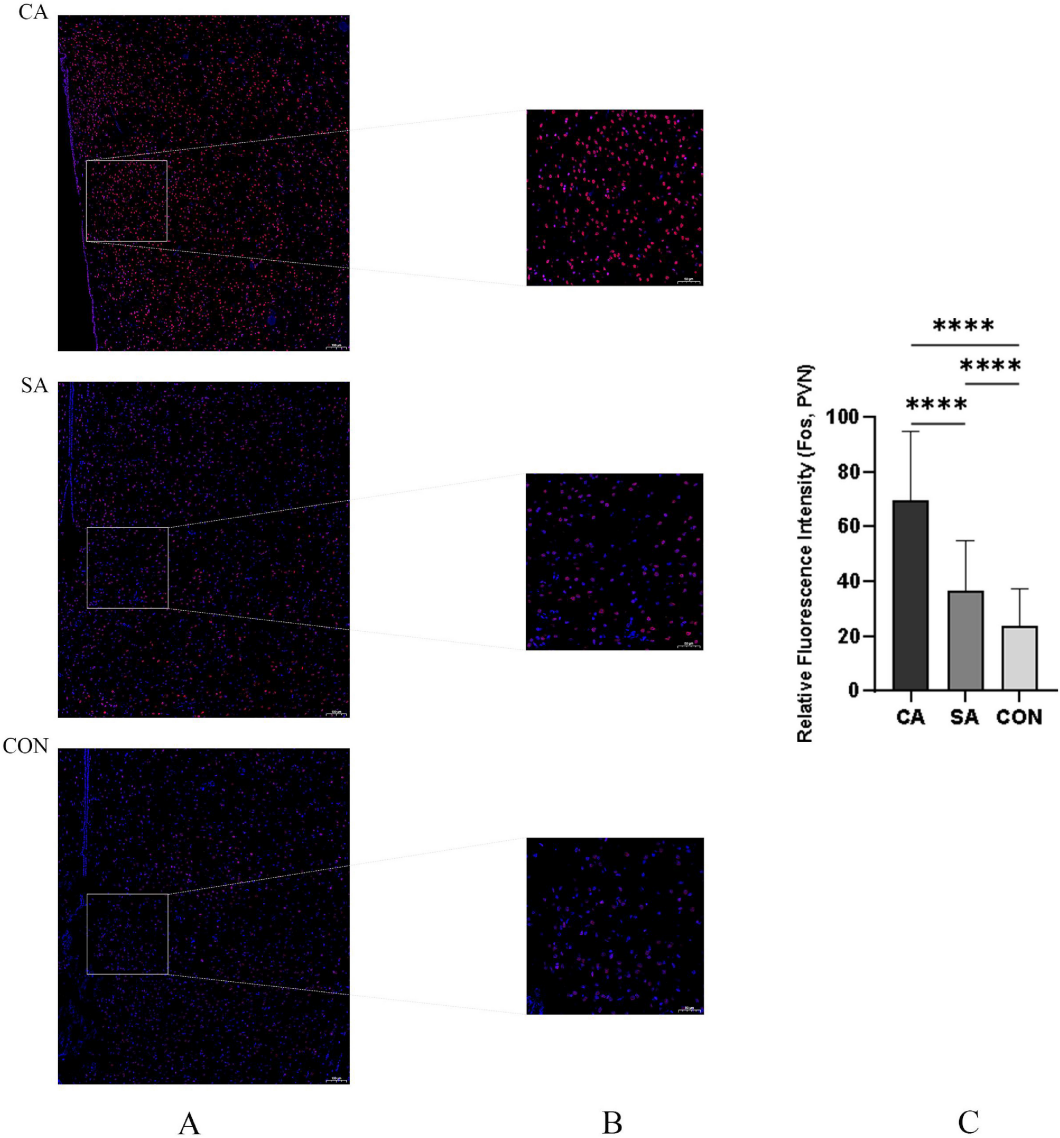
Immunofluorescence quantification of Fos-positive cells in the citric acid (CA), saline control (SA), and blank control (CON) groups. **(A)** Low-magnification (×10, air objective) immunofluorescence overview showing the anatomical location of c-Fos-positive neurons (red) in the PVN region of CA, SA, and CON groups. Images were acquired using a Zeiss LSM 880 laser scanning confocal microscope. Scale bar: 100 μm. **(B)** Representative immunofluorescence images showing c-Fos-positive neurons (red) in the PVN of CA, SA, and CON groups. Scale bar: 50 μm. **(C)** Quantitative analysis of c-Fos expression levels in the PVN. The histogram shows the relative fluorescence intensity of c-Fos signals, measured as mean optical density and normalized to the background. Data are presented as mean ± SEM (*n* = 6 per group). ****P < 0.0001 vs. SA and CON groups (one-way ANOVA followed by Tukey’s *post-hoc* test).

**FIGURE 10:**
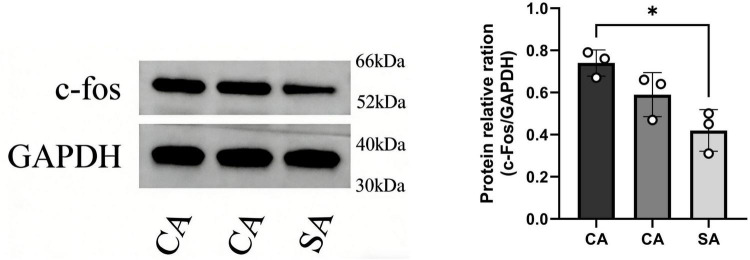
Western blot analysis showing protein bands of the target molecule (c-Fos) and internal reference (GAPDH) with their relative expression levels in the CA and SA groups (*P < 0.05 for CA vs. SA).

Immunofluorescence staining showed that c-Fos protein (a marker of neuronal activation) was predominantly localized in the nuclei of neurons, appearing as bright red fluorescence. Semi-quantitative analysis revealed that the relative fluorescence intensity of c-Fos protein in the PVN region of the CA group was significantly higher than that of SA and CON (*P* < 0.001) (Figure 9). Western blot analysis further confirmed that the expression level of c-Fos protein in the PVN of the CA group was markedly increased (Figure 10). Statistical analyses were performed using the animal as the experimental unit (*n* = 6 per group). These findings indicate increased neuronal activation in the PVN under cough hypersensitivity conditions; however, the functional role of these neurons requires further investigation.

#### 3.3.2 Increased SP expression

**FIGURE 11:**
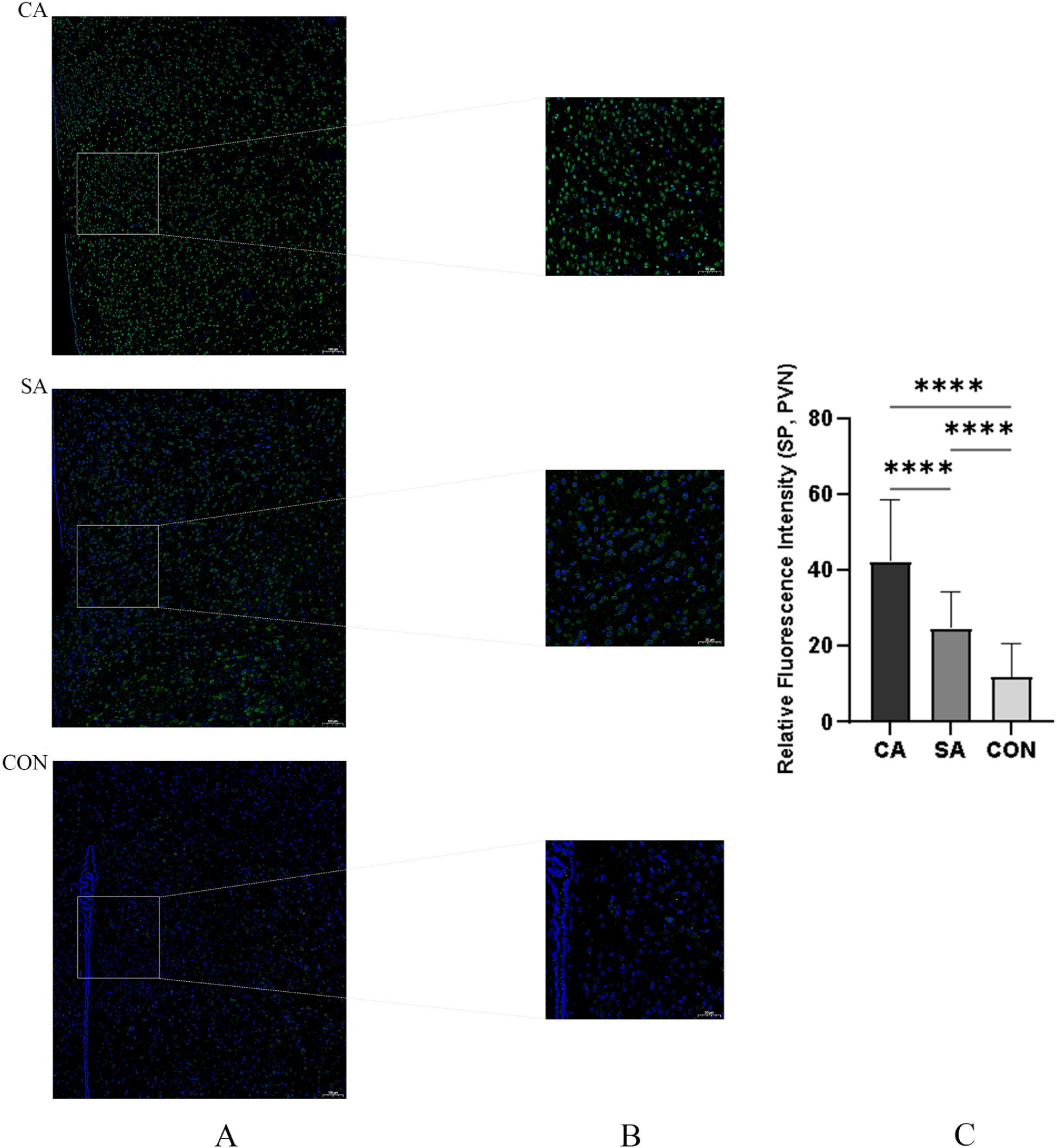
Comparison of substance P (SP)-positive cell counts in the citric acid (CA), saline control (SA), and blank control (CON) groups. **(A)** Low-magnification (×10, air objective) immunofluorescence overview showing the anatomical location of SP-positive neurons (green) in the paraventricular nucleus (PVN) region of CA, SA, and CON groups. Images were acquired using a Zeiss LSM 880 laser scanning confocal microscope. Scale bar: 100 μm. **(B)** Representative immunofluorescence images showing SP-positive neurons (green) in the PVN of CA, SA, and CON groups. Scale bar: 50 μm. **(C)** Quantitative analysis of SP expression levels in the PVN. The histogram shows the relative fluorescence intensity of SP signals, measured as mean optical density and normalized to the background. Data are presented as mean ± SEM (*n* = 6 per group). ****P < 0.0001 vs. SA and CON groups (one-way ANOVA followed by Tukey’s *post hoc* test).

Substance P immunofluorescence signals appeared green and were widely distributed in the cytoplasm and processes of neurons. Statistical analyses were performed using the animal as the experimental unit (*n* = 6 per group). Semi-quantitative analysis showed that the relative fluorescence intensity of SP in the PVN of the CA group was significantly higher than that of SA and CON, approximately 1.5-fold higher than that of the SA group (*P* < 0.001; Figure 11), indicating that SP–mediated signaling in the PVN was markedly activated during cough hypersensitivity.

#### 3.3.3 Significant increase in Fos/SP double-positive neurons

**FIGURE 12:**
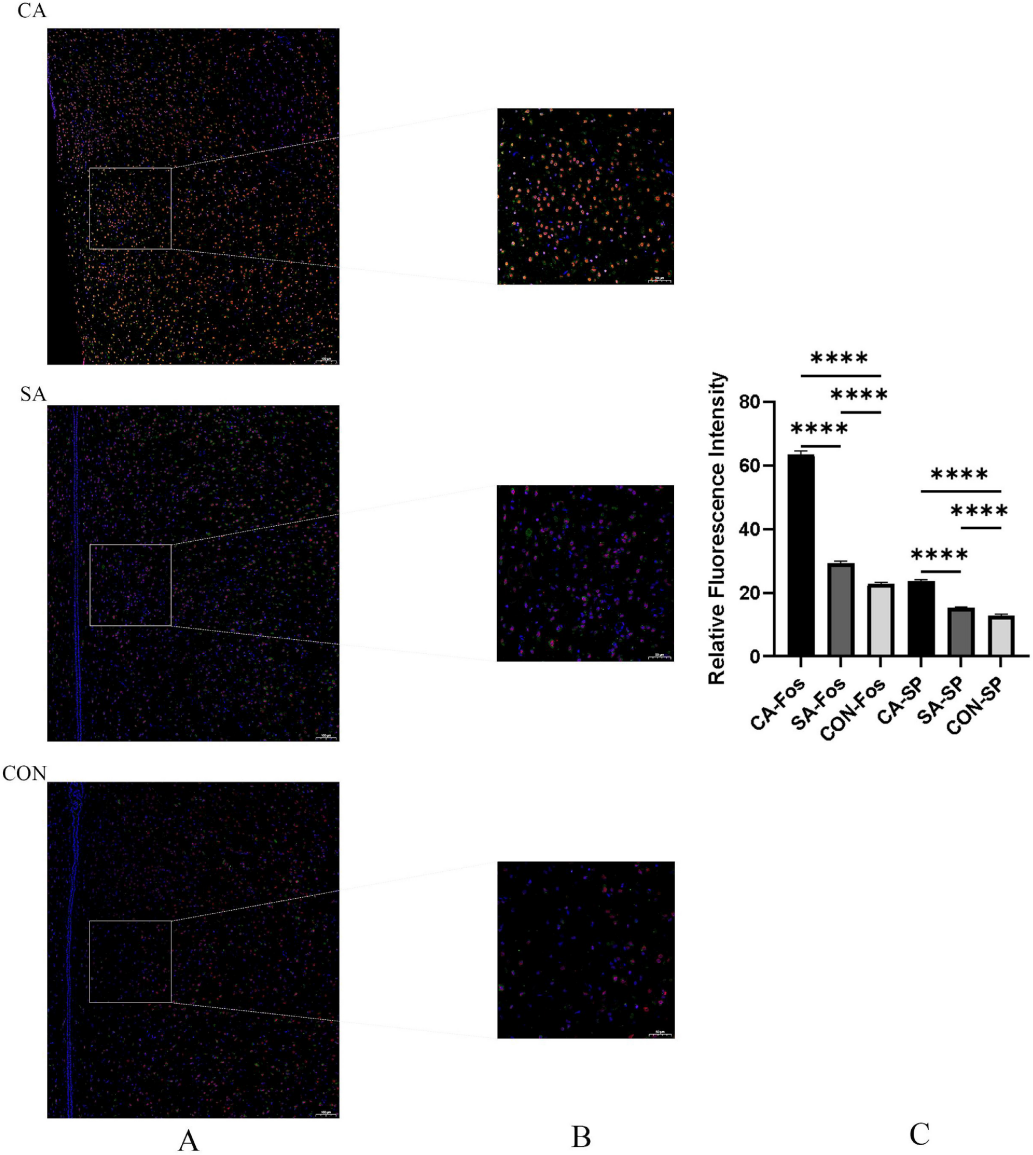
SP/Fos double-positive neurons in the paraventricular nucleus (PVN) of the citric acid (CA), saline control (SA), and blank control (CON) groups. **(A)** Low-magnification (× 10, air objective) multichannel confocal microscopy images of the PVN region. The images show the anatomical localization of neurons, with substance P (SP) (green fluorescence) and c-Fos (red fluorescence) signals. Scale bar: 100 μm. Images were acquired using a Zeiss LSM 880 laser scanning confocal microscope. **(B)** Representative immunofluorescence images showing SP/Fos double-positive neurons (yellow, due to colocalization of red and green signals), SP-single positive neurons (green), and Fos-single positive neurons (red). Scale bar: 50 μm. **(C)** Quantitative analysis of SP and Fos expression levels in the PVN. The histogram shows the relative fluorescence intensity of SP and Fos signals in the CA, SA, and CON groups, measured as mean optical density and normalized to the background. Data are presented as mean ± SEM (*n* = 6 per group). ****P < 0.0001 vs. SA and CON groups (one-way ANOVA followed by Tukey’s *post-hoc* test).

Multichannel confocal microscopy revealed more SP/Fos double-positive neurons in the PVN of the CA group than in controls (Figure 12). Colocalization analysis showed that these double-positive neurons accounted for 66.17% ± 3.20 of all SP-positive neurons (*P* < 0.001), suggesting that SP-mediated signaling in the PVN may play a key role in regulating cough hypersensitivity.

### 3.4 PVN–airway neural connection

**FIGURE 13:**
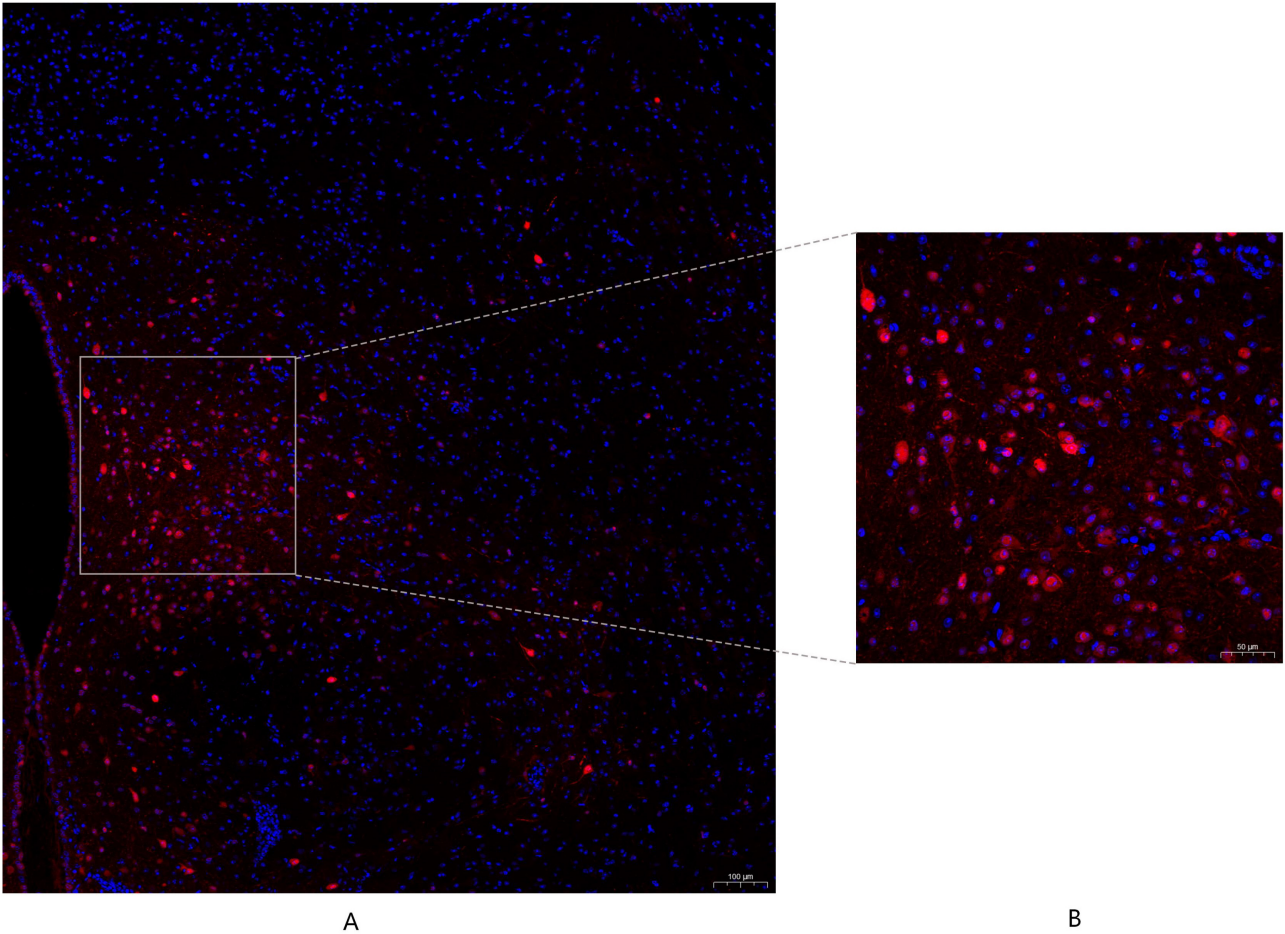
TdTomato-labeled neurons in the paraventricular nucleus (PVN) following HSV-tdTomato retrograde tracing. **(A)** Low - magnification (×10, air objective) confocal microscopy image of the PVN region acquired using a Zeiss LSM 880 laser scanning confocal microscope. This image shows the anatomical localization of neurons, including tdTomato labeled neurons (red fluorescence, indicating retrogradely labeled PVN neurons). Scale bar: 100 μm.; **(B)** High - magnification (×20, oil immersion objective) representative immunofluorescence image of the boxed area in panel **(A)**, showing tdTomato - labeled Substance P (SP) - positive neurons (red). Scale bar: 50 μm.

To investigate whether the PVN participates in airway regulation via direct neural pathways, we employed HSV-tdTomato retrograde tracing. At 72 h post-viral injection into the tracheal wall, numerous tdTomato-labeled neurons were detected in the PVN (Figure 13), providing morphological evidence suggestive of a neural pathway linking the PVN to the airway, although the transsynaptic nature of the tracer precludes confirmation of a direct monosynaptic connection.

### 3.5 Proposed mechanistic framework of PVN–airway pathway in cough hypersensitivity

**FIGURE 14:**
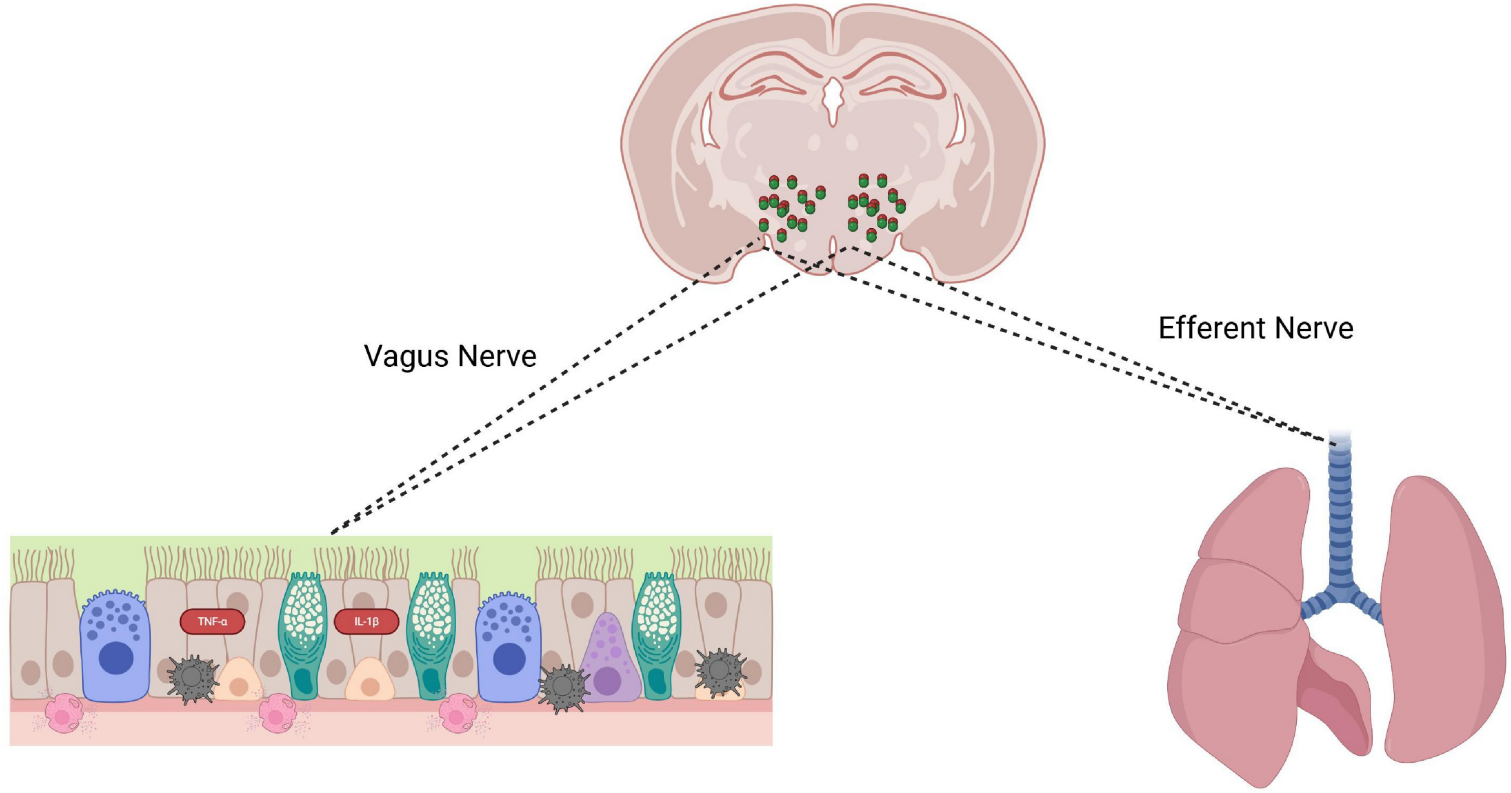
Proposed mechanistic framework of the paraventricular nucleus (PVN)–airway neural pathway in cough hypersensitivity.

Based on the above results, we constructed a mechanistic model illustrating how the PVN regulates cough hypersensitivity (Figure 14). Chronic airway inflammation activates SP-expressing PVN neurons via peripheral sensory afferents. These activated PVN neurons may modulate airway function via SP-mediated signaling, exacerbating neurogenic airway inflammation and sensitizing sensory nerve endings. This creates a periphery–central–periphery positive feedback loop promoting cough hypersensitivity.

## 4 Discussion

In this study, we developed a stable citric acid–induced cough hypersensitivity model in juvenile guinea pigs and applied a comprehensive set of approaches—including behavioral assessment, histopathological examination, neurochemical analysis, and viral tracing, to systematically elucidate the critical role of the PVN in chronic cough hypersensitivity. The major findings are as follows: (1) repeated citric acid exposure consistently induced a stable cough hypersensitivity phenotype; (2) under hypersensitive conditions, PVN neurons were markedly activated, with significant upregulation of SP expression and a notable increase in the proportion of SP/Fos double-positive neurons; and (3) retrograde tracing provided direct morphological evidence of a neural connection between the PVN and the airway, indicating that PVN SP-expressing neurons may participate in cough sensitization through a central-airway neural pathway.

### 4.1 Model development and validation

Chronic cough is a common pediatric respiratory symptom caused by abnormal elevation of cough reflex sensitivity. Because children’s respiratory and nervous systems are immature, cough regulation exhibits age-specific features. Thus, developing clinically relevant juvenile animal models is crucial. We used 3-week-old guinea pigs to model this childhood developmental stage (Lucanska et al., 2020). We used repeated citric acid nebulization for modeling. Its advantage lies in the continuous activation of TRPV1 receptors on airway C and Aδ fibers, thereby mimicking the peripheral sensory nerve sensitization associated with chronic airway inflammation rather than acute injury. This approach mimics certain aspects of airway sensory sensitization associated with chronic cough (Farmer et al., 2026).

For model validation, we utilized a multidimensional assessment strategy. Behaviorally, cough frequency exhibited a time-dependent increase with repeated stimulation. Functionally, capsaicin challenge testing demonstrated a significant reduction in the cough threshold (C5) (Jiang et al., 2019). Histopathologically, HE and PAS staining revealed characteristic features of neurogenic airway inflammation and remodeling, consistent with the pathological changes seen in clinical chronic cough (Ruyle et al., 2023). Critically, open-field testing ruled out potential confounding effects of general locomotor activity on cough behavior, thereby ensuring the specificity of the observed responses. This integrated validation system not only confirmed the successful establishment of the model but also highlighted its persistent cough hypersensitivity coexisting with airway inflammation, a phenotype closely resembling that of pediatric chronic cough patients. Accordingly, the model serves as a reliable and clinically relevant experimental platform for subsequent studies on central regulatory mechanisms.

It should be noted that citric acid is a strong airway irritant that induces robust cough reflex responses primarily through activation of TRPV1 receptors on airway epithelial cells and vagal sensory afferents, including C fibers and Aδ fibers (Poliacek et al., 2017). Although repeated exposure to citric acid can induce persistent coughing behavior and increased cough sensitivity in animal models, this paradigm largely reflects reflex coughing triggered by strong irritant stimuli rather than the cough hypersensitivity state commonly observed in patients with chronic cough (Mei et al., 2022). In clinical settings, cough hypersensitivity is often characterized by exaggerated responses to otherwise innocuous stimuli, such as cold air, talking, or cooking fumes (Jiménez-Gómez, 2026). Therefore, the central nervous system changes observed in this study, including PVN activation, may partly reflect sustained activation driven by strong peripheral sensory inputs rather than fully representing the mechanisms of central sensitization underlying clinical chronic cough. Future studies incorporating low-threshold stimuli or environmental triggers may further improve the translational relevance of experimental cough hypersensitivity models.

### 4.2 PVN–airway pathway in cough sensitization

Traditionally, research on the central regulation of cough has been focused mainly on brainstem circuits (Iremonger and Power, 2025; Savić et al., 2022). A key finding is extending central cough regulation from the brainstem to the hypothalamus, a higher-order integrator. The PVN, serving as a primary hub for autonomic and neuroendocrine integration, receives widespread visceral sensory inputs and sends descending regulatory commands. Using retrograde HSV tracing—a key morphological tool (Cinelli et al., 2023; Lu et al., 2024)—we provide evidence for a neuroanatomical link between PVN and the airway. Given the transsynaptic capability of the tracer used, the observed connectivity suggests a potential pathway through which the PVN may influence airway function. During cough hypersensitivity, many PVN neurons were activated, as shown by increased c-Fos expression. This functional evidence, combined with the aforementioned morphological findings, collectively supports the hypothesis that the PVN-airway neural pathway may be involved in cough central sensitization. These findings challenge the traditional view that cough sensitization originates solely from brainstem circuits, providing robust experimental evidence and novel insights for understanding the brain-lung axis regulation of chronic cough.

### 4.3 SP-expressing PVN neurons in central cough sensitization

Substance P is known to mediate peripheral neurogenic inflammation and cough sensitivity, but its regulatory mechanisms within the CNS are poorly understood (Liu et al., 2025; Tenorio-Lopes and Kinkead, 2021). In the present study, SP expression in the PVN was significantly elevated in cough-hypersensitive juvenile guinea pigs. More importantly, double-label immunofluorescence for SP and Fos revealed that as many as 66.17% of SP-positive neurons were also Fos-positive, suggesting that SP-expressing neurons in the PVN were recently activated.

Unlike previous studies that primarily focused on peripheral mechanisms, the present study suggests a potential central sensitization mechanism: airway inflammatory signals ascend to activate SP-expressing neurons within the PVN, which could subsequently engage descending neural pathways influencing airway function, potentially through SP-mediated signaling or related effector pathways, further aggravating peripheral nerve sensitization and sustaining cough hypersensitivity. However, given that Fos expression only indicates recent activation and does not define functional involvement, this proposed loop requires direct experimental validation. This discovery expands the theoretical framework of the brain–lung axis in chronic cough regulation to the central nervous system level and provides new experimental evidence for understanding the mechanisms of central sensitization.

Nevertheless, although SP and neurokinin-1 (NK1) receptor pathways have been implicated in experimental cough models, clinical studies targeting this pathway have shown limited efficacy. The NK1 receptor antagonist Orvepitant failed to demonstrate consistent antitussive benefits in patients with chronic cough, and earlier peripherally acting NK1 antagonists similarly showed no robust clinical effects (Badri and Smith, 2019; Smith et al., 2020). These findings suggest that SP signaling alone may not be sufficient to drive cough hypersensitivity in humans.

### 4.4 Clinical implications and translational potential

We propose a closed-loop model where peripheral inflammation activates SP-expressing PVN neurons, which exert descending control to drive cough hypersensitivity. This model provides a novel framework for understanding central mechanisms of persistent chronic cough. Given the limited efficacy and side effects of current antitussives, our findings suggest that PVN SP-mediated signaling is a promising target for modulating central cough sensitization, informing novel central-targeted antitussive strategies.

This study has certain limitations: firstly, our findings were obtained from an animal model, and their applicability to pediatric chronic cough needs to be further validated in clinical samples; second, although our data suggest a potential role for the PVN SP pathway in cough hypersensitivity, the current evidence remains largely correlative. Future studies could incorporate optogenetics, chemogenetics, or specific receptor antagonists to enable precise modulation of SP-expressing neurons in the PVN. Such approaches would help clarify the causal role of PVN SP neurons in both the initiation and maintenance of cough hypersensitivity, thereby providing experimental support for the development of novel centrally targeted antitussive therapies. Third, in the Western blot analysis of c-Fos protein shown in Figure 9, only the CA and SA groups were compared, and no CON group was included. Although this design verifies the specific neuronal activation in the CA group relative to the SA group, it does not provide a comparison with an untreated baseline. This limitation should be considered when interpreting the molecular data, and future studies should include a CON group to provide a more comprehensive evaluation. Finally, the PVN is a multifunctional integrative center involved in autonomic regulation, neuroendocrine responses, and stress signaling. Therefore, PVN activation observed in this study may also reflect broader physiological responses to airway irritation rather than exclusively cough-specific neural processing.

## 5 Conclusion

This study indicates that activation of SP-expressing neurons in the PVN is associated with chronic cough hypersensitivity, possibly involving a PVN-airway neural pathway. Using a juvenile guinea pig model, we found that repeated airway stimulation induced persistent cough hypersensitivity with significant airway inflammation. Immunofluorescence revealed increased SP/Fos co-expression in PVN neurons, indicating their activation during cough hypersensitivity. HSV retrograde tracing suggested the existence of PVN-airway projections, providing anatomical evidence for this neural circuit. These findings extend cough regulation mechanisms to the hypothalamic level and support further investigation of PVN SP neurons as a potential central target for cough treatment.

## Data Availability

The original contributions presented in this study are included in the article/supplementary materials, further inquiries can be directed to the corresponding authors.
